# Functional characterization of a loss-of-function mutant I324M of arginine vasopressin receptor 2 in X-linked nephrogenic diabetes insipidus

**DOI:** 10.1038/s41598-021-90736-z

**Published:** 2021-05-26

**Authors:** Lixia Wang, Weihong Guo, Chunyun Fang, Wenli Feng, Yumeng Huang, Xiaona Zhang, Ming Liu, Jingqiu Cui

**Affiliations:** Department of Endocrinology and Metabolism, Tianjin Medical University General Hospital, Tianjin, 300052 China

**Keywords:** Acid, base, fluid, electrolyte disorders, Hormone receptors, Endocrine system and metabolic diseases

## Abstract

X-linked nephrogenic diabetes insipidus (X-linked NDI) is a rare inherited disease mainly caused by lost-of-function mutations in human *AVPR2* gene encoding arginine vasopressin receptor 2 (V2R). Our focus of the current study is on exploration of the functional and biochemical properties of Ile324Met (I324M) mutation identified in a pedigree showing as typical recessive X-linked NDI. We demonstrated that I324M mutation interfered with the conformation of complex glycosylation of V2R. Moreover, almost all of the I324M-V2R failed to express on the cell surface due to being captured by the endoplasmic reticulum control system. We further examined the signaling activity of DDAVP-medicated cAMP and ERK1/2 pathways and the results revealed that the mutant receptor lost the ability in response to DDAVP stimulation contributed to the failure of accumulation of cAMP and phosphorylated ERK1/2. Based on the characteristics of molecular defects of I324M mutant, we selected two reagents (SR49059 and alvespimycin) to determine whether the functions of I324M-V2R can be restored and we found that both compounds can significantly “rescue” I324M mutation. Our findings may provide further insights for understanding the pathogenic mechanism of *AVPR2* gene mutations and may offer some implications on development of promising treatments for patients with X-linked NDI.

## Introduction

Arginine vasopressin receptor 2 (V2R) is encoded by the *AVPR2* gene located on the Xq28 chromosome region, which belongs to the seven transmembrane-domain G protein-coupled receptor (GPCR)^1^. The newly synthesized V2R was firstly modified with core N-linked glycosylation in the ER, and then completed complex-N-linked and O-linked glycosylation modification in Golgi network^2^, eventually become the mature form with 371 amino acids presenting on the plasma membrane. V2R is expressed in the renal tubules, predominantly in the collecting ducts, where it mainly plays a role in promoting water reabsorption and urine concentration under the administration of arginine vasopressin (AVP). The latter also called antidiuretic or vasoconstrictor, is a 9-peptide hormone, mainly synthesized by the supraoptic nucleus of the hypothalamus and stored in the posterior pituitary gland^3, 4^. Once the body’s osmotic pressure increases or blood volume is insufficient, AVP will be released from the posterior pituitary and binding to V2R inserted into the plasma membrane. AVP-V2R complex catalyzes the release of αs-GTP from the receptor-coupled stimulatory G protein (Gs), and activates adenylate cyclase (AC), followed by the increasement of the cyclic adenosine monophosphate (cAMP) concentration and activation of protein Kinase A (PKA). Then the translocation of aquaporin 2 (AQP2) was initiated from the intracellular storage vesicles pool to the apical plasma membrane for maintaining kidney water homeostasis^5–7^. Besides its classical biological effects, V2R can also initiate a mitogen-activated protein kinase (MAPK) cascade independent of the G protein, phosphorylating extracellular-regulated protein kinase (ERK1/2) and thus promoting the translocation of AQP2^8–10^. Mutations of V2R or AQP2 will disrupt the balance of AVP-V2R-AQP2 system, producing excess diluted urine excreting from kidney.

Congenital nephrogenic diabetes insipidus (CNDI) is characterized by the inability of kidney to concentrate urine, even normal or increased plasma AVP levels. The clinical manifestations in patients who suffered from CNDI include polyuria, polydipsia, hypernatremia and hypobaric urine^11, 12^. Mutations of *AVPR2* and *AQP2* gene are the pathogenic basis of CNDI. Up to 90% of the CNDI occurring due to mutations of *AVPR2* gene, the remaining approximately 10% of cases are autosomal recessive or dominant genetic disease caused by mutations of *AQP2* gene^2, 13^. The different V2R mutant cellular biological behaviors correlate with the broad spectrum of clinical manifestation, ranging from classical NDI symptoms to nephrogenic syndrome of inappropriate diuresis. Up to now, at least 287 *AVPR2* gene mutations have been reported, including missense/nonsense mutations, small/ large fragment deletion, splicing site mutations and etc*.*, 177 of them are missense mutations, accounting for nearly 62%^5, 14^. A few of gain-of-function mutations, such as R137C, R137L I130N and F229V that cause constitutive activation of receptors, leading to X-linked nephrogenic syndrome of inappropriate antidiuresis (NSIAD)^15, 16^, while most of *AVPR2* gene mutations are loss-of-function that cause X-linked nephrogenic diabetes insipidus (X-linked NDI)^7, 17, 18^. The reasons of these inherited defects caused by mutations in the *AVPR2* gene can be boiled down to the following five classes, I: Impairment of mRNA processing and stability. II: Mutant receptors are misfolded and caught by ER quality control system. III: An inability of AVP-V2R binding although V2R maturation and trafficking are not affected. IV: Mature V2R has successfully expressed on cell surface but the ability of V2R couple to Gs protein is disturbed. V: The misrouting expression of receptors causes AVP-independent constitutive desensitization and internalization. To date, the type II is the most common cause by which mutant V2R fails to work properly^19^. So more and more pharmacological chaperones (SR121463, SR49059, VPA985, OPC31260 and so on), a class of small molecular compounds with pharmacological activity targeting to promote protein folding correctly, were used to increase the export of V2R captured by the ER to the cell surface^20^. In addition, alvespimycin, a class of chemically unrelated compounds that as an inhibitor of heat-shock protein 90 (HSP90), has been recently reported that they can up-regulate the expression of wild type V2R on the cell surface and increase the activity of its downstream cAMP signaling pathway^21^.

Recently, some of the reported mutations have been studied in detail, however, the roles of many mutations are still not been elucidated. We have previously reported a pedigree carrying *AVPR2* missense mutation (c.972C > G; p. I324M)^22^. In present study, we not only focused on studying the molecular biological behaviors of I324M-V2R terms of its maturation, trafficking, stability and DDAVP-mediated signaling properties including cAMP and ERK1/2 cascade, but also exploring the potential treatments for patients carrying I324M mutation by using SR49059 (a V_1a_ receptor antagonist) and alvespimycin. The results demonstrated that I324M mutation causes X-linked NDI by disrupting maturation and transportation of the receptor, followed by the inactivation of V2R-mediated signaling pathways. Furthermore, we confirmed that the normal functions of I324M-V2R could be partially restored by SR49059 and alvespimycin.

## Results

### I324M mutant impaired the V2R maturation and expression on the plasma surface

V2R is a glycoprotein receptor with a glycosylation site at position 22. Glycosylation is one of the most important post-translational modification, which plays an essential role in maintaining the structure and function of proteins. Therefore, identifying the degree of glycosylation of glycoproteins is important for understanding the pathogenic basis of V2R mutants. Endoglycosidase H (EndoH), peptide-N-glycosidase F (PNGase.F) and O-glycosidase & Sialidase can cleave N-linked high mannose groups, all N-linked sugar moieties and O-linked disaccharides respectively^23^. The maturity of this receptor can be assessed according to the different sensitivity of V2R to the deglycosylases as mentioned above^24^.

**Figure 1:**
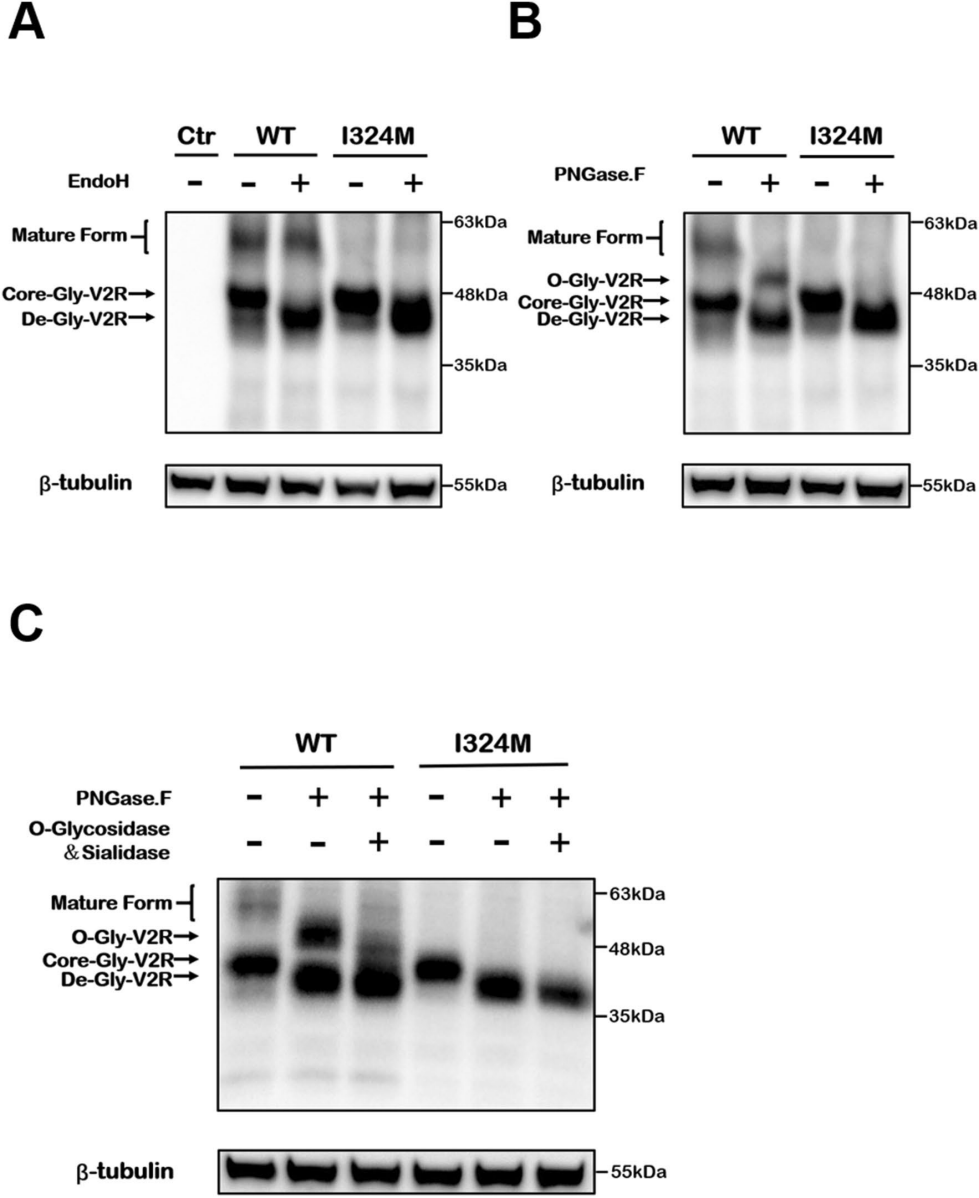
HEK293T cells expressing the I324M-V2R fail to produce mature receptors, which was in the form of immature precursors without complex glycosylated groups. HEK293T cells transiently transfected with cDNAs encoding the pcDNA3.1 (Ctr), wild type-V2R (WT) and I324M-V2R (I324M). 48 h after transfection, the cells lysate was treated with or without EndoH, PNGase.F, or O-glycosidase & Sialidase and detected by western blotting using anti-Myc rabbit polyclonal antibody. (**A**) Lanes 1 was used as negative control. Lanes 2–3 and 4–5 showed the EndoH sensitivity of WT group and I324M group respectively. (**B**) Lanes 1–2 showed the PNGase.F sensitivity of WT group; lanes 3–4 showed the PNGase.F insensitivity of I324M group. (**C**) Lane 1–3 showed the PNGase.F and O-glycosidase & Sialidase sensitivity of WT group; lanes 4–6 showed the PNGase.F and O-glycosidase & Sialidase insensitivity of I324M group. EndoH: endoglycosidase H; PNGase.F: peptide-N-glycosidase F. Representative of 3 separate experiments.

In order to detect the receptor maturation state of WT-V2R and I324M-V2R, HEK293T cells expressing WT-V2R and I324M-V2R were treated with or without EndoH, PNGase.F or O-glycosidase & Sialidase before analysis with western blotting. As shown in Fig. 1A, the group of WT-V2R was detected two main bands compared with control (Ctr) (Fig. 1A, compare lanes 1 and 2), one at ~ 48 kDa that represented the core glycosylated receptors (Core-Gly-V2R) and the other at ~ 63 kDa, which was thought to be the mature form of V2R with fully glycosylated modification. When the WT-V2R group was pretreated with Endo H, the position of the ~ 63 kDa band was not altered whereas ~ 48 kDa band was removed to ~ 42 kDa, which was described as de-glycosylated V2R (De-Gly-V2R) (Fig. 1A, compare lanes 2 and 3). EndoH resistance of the ~ 63 kDa form suggested that a considerable amount of V2R had been transported from the ER to Golgi apparatus or cell surface, so after processing with PNGase, F (Fig. 1B, compare lanes 1 and 2), we found that the band was removed to ~ 52 kDa and could be further dropped to ~ 42 kDa using O-glycosidase & Sialidase in the WT-V2R group (Fig. 1C, compare lanes 1, 2 and 3). In contrast to WT-V2R, I324M-V2R only formed a band at 48 kDa without the upper mature glycosylated band (Fig. 1A, compare lanes 2 and 4), but the sensitivity to EndoH was as same as in the WT-V2R group, revealing the process of immature glycosylation in ER (Fig. 1A, compare lanes 3 and 5). Furthermore, I324M-V2R also failed to form PNGase.F-sensitive band (Fig. 1B, compare lanes 3 and 4), confirming that the mutation did substantially impair receptor maturation, which was further illustrated by the disappearance of O-glycosylation moieties (Fig. 1C, compare lanes 4, 5 and 6). These findings confirmed that most, if not all, I324M-V2R were exist in an immature form.

### I324M mutant hindered the transport of V2R, making almost all receptors trapped in the intracellular compartments

**Figure 2:**
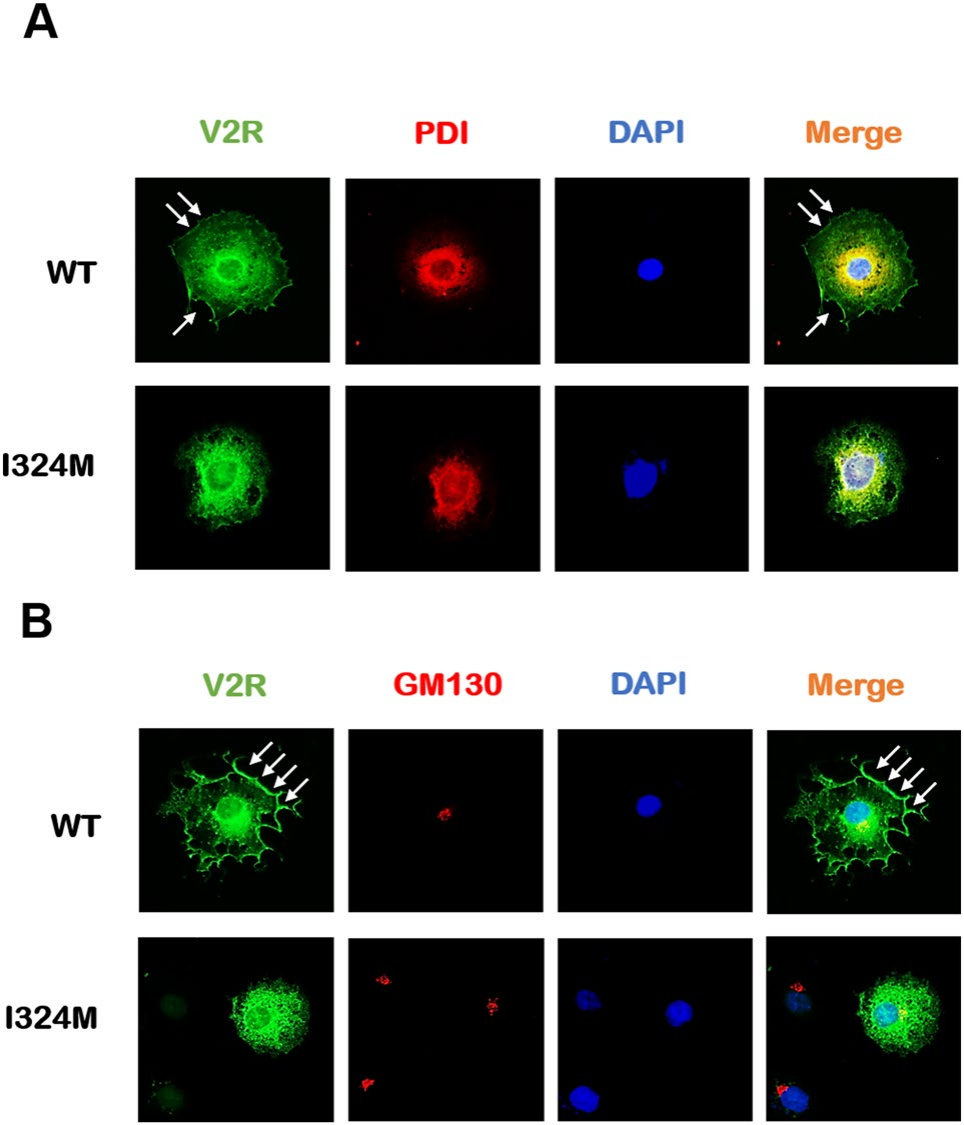
The V2R proteins carrying I324M mutant lost the ability expressed on the plasma membrane and almost entirely retained in the ER. COS7 cells were transiently expressed Myc-tagged-WT-V2R (WT) or Myc-tagged-I324M-V2R (I324M), respectively. (**A**) The fluorescence imaging of ER location of WT and I324M co-stained with PDI respectively using anti-Myc rabbit polyclonal antibody and anti–PDI mouse monoclonal antibody. (**B**) The fluorescence imaging of Golgi location of WT and I324M co-stained with GM130 respectively using anti-Myc rabbit polyclonal antibody and anti–GM130 mouse monoclonal antibody. V2R was shown in green; PDI and GM130 were shown in red; The superposition of V2R and PDI or GM130 was shown in orange. PDI: protein disulfide isomerase, a marker of endoplasmic reticulum (ER); GM130: a marker of Golgi apparatus; DAPI: a marker of nucleus. Representative of 3 separate experiments.

As mentioned before, I324M-V2R mutation blocked the protein proper glycosylation resulting in the dysmaturity of V2R. Mature glycosylated modification is essential for the transposition of glycoproteins from ER to Golgi apparatus and targeting to the cell surface. Therefore, whether the immaturity of V2R-I324M has a potential influence on its intracellular distribution was the focus of our further research. Immunofluorescence technology was applied to compare the trafficking difference of the WT-V2R and I324M-V2R. COS7 cells were used to present the receptor’s subcellular localization because of its prominent membrane structure that makes it easy to distinguish the specific location of V2R proteins. WT-V2R and I324M-V2R were co-stained with protein disulfide isomerase (PDI) and Golgi marker (GM130), which are used as the markers of endoplasmic reticulum (ER) and Golgi apparatus, respectively^25^. As shown in Fig. 2A, WT-V2R was predominantly localized on the plasma membrane as pointed by the white arrows (Fig. 2A, the upper row). Furthermore, we can also note that a small part of receptors was co-located with PDI may be the result of the characteristics of COS7 cell line and the application of transient transfection technology. As expected, COS7 cells expressing I324M-V2R were barely detected fluorescence in cell surface, but almost completely co-stained with PDI (Fig. 2A, the lower row). The performance of co-staining with GM130 is roughly similar to that of Fig. 2A, the difference in the fluorescence density of the receptor on plasma membrane between WT-V2R and I324M-V2R was still obvious (Fig. 2B, the upper row and lower row). That is to say, I324M-V2R mostly retained in intracellular regions compared with V2R-WT. These results indicated that I324M mutant was unable to target on the cell surface to perform a function, because of the receptor transport pathway disordered.

### I324M mutant did not affect the production and stability of V2R proteins

**Figure 3:**
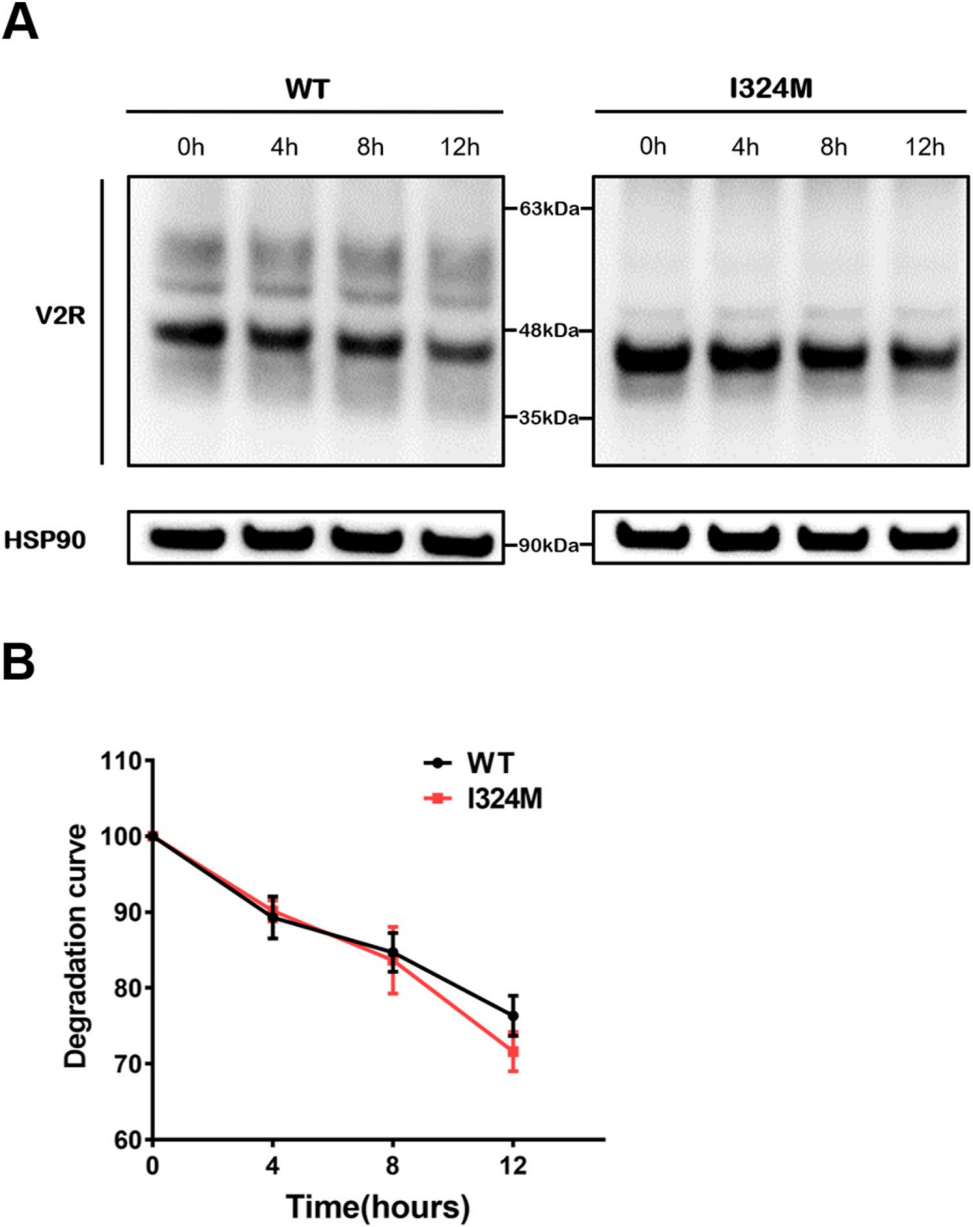
The I324M mutant have no obvious influence on V2R protein’s production and stability compared with WT. (**A**) HEK293T cells transiently expressing either WT-V2R (WT) or I324M-V2R (I324M), 48 h after transfection, cells were incubated with 20 μg/ml cycloheximide (CHX) for 0 ,4 ,8 and 12 h before detected by western blotting using anti-Myc rabbit polyclonal antibody. (**B**) Density of degraded protein bands were normalized against endogenous HSP90 and quantified by ImageJ 1.49 (https://imagej.nih.gov/ij/) The data was from four independent experiments.

Most V2R mutations result in protein retention in the endoplasmic reticulum (ER) and may trigger the endoplasmic reticulum-associated degradation (ERAD) pathway, thereby affecting the stability of the receptor. Therefore, the cycloheximide (CHX) chase assay was performed to test the protein stability in the groups of WT-V2R and I324M-V2R. As shown in Fig. 3A and B, we could see the degradation rate of I324M-V2R group was almost identical with WT-V2R group after treated with CHX for 4, 8 h. Although it looks like there was a difference in treatment with CHX for 12 h, unfortunately, the results had no statistical difference. Moreover, we can also observe that the production of V2R proteins in I324M-V2R was similar to WT-V2R (Fig. 3A). Consequently, these results illuminated that I324M mutation had no influence on the stability and total protein amount of V2R.

### I324M mutant inactivated G protein-dependent cAMP signaling activity of V2R under the stimulation of DDAVP

**Figure 4:**
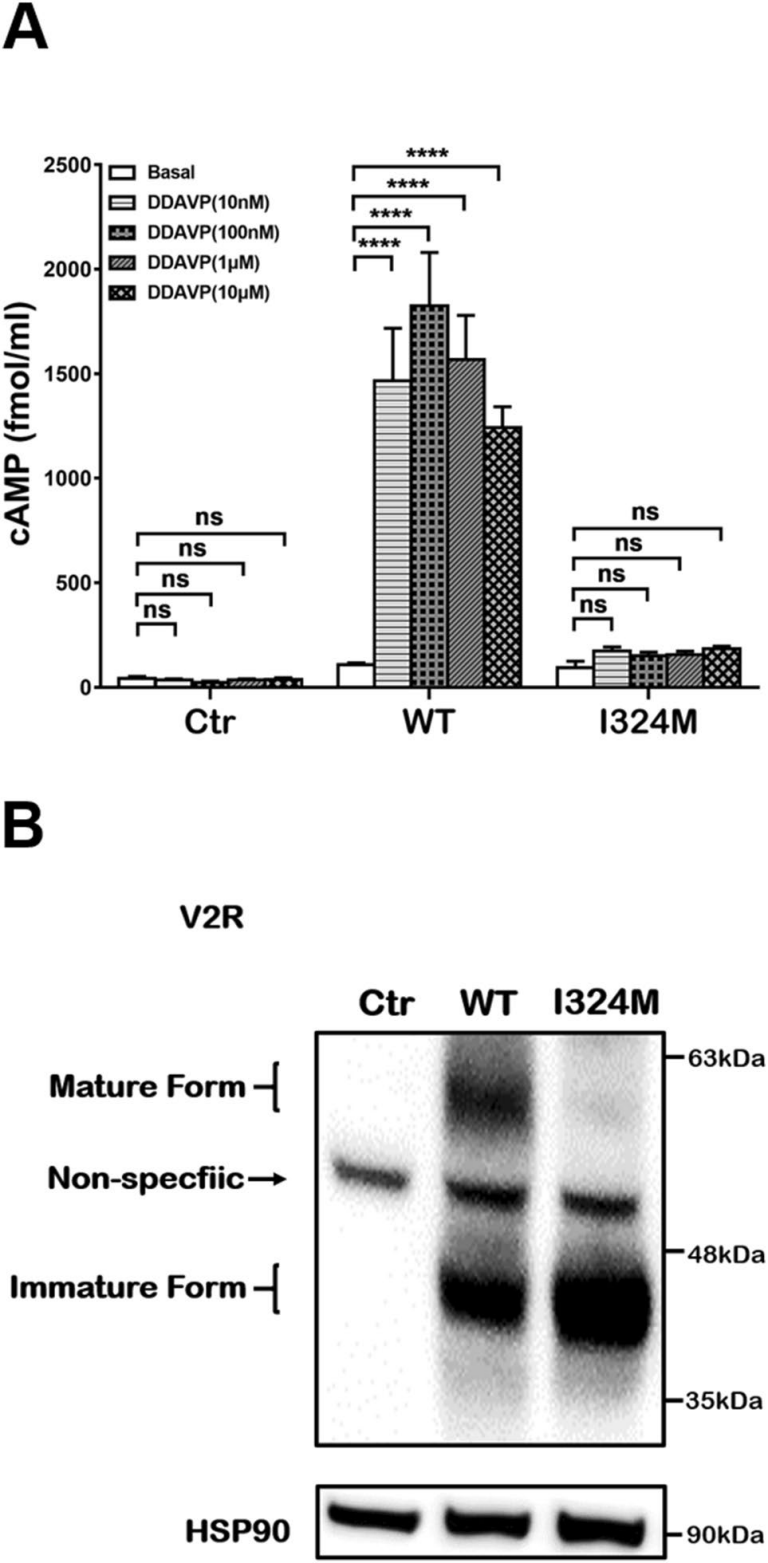
There is no increasement in cAMP accumulation of cells expressing I324M mutant under the stimulation of DDAVP. HEK293T cells were transiently transfected with pcDNA3.1 (Ctr), WT-V2R (WT) or I324M-V2R (I324M). cAMP measurements were performed after treated with or without various concentrations of DDAVP (1 nM, 100 nM, 1 μM or 10 μM) for 15 min (as described in “Methods”). (**A**) Results were shown as bar graph and data represent mean ± SEM using Two-way ANOVA were obtained from three independent experiments. ****P < 0.0001. (**B**) V2R expression of WT and I324M groups in the absence of DDAVP. Representative of 3 separate experiments.

The normality of cAMP production in response to AVP stimulation is the key for kidney to maintain body’s water balance. Therefore, we monitor basal or DDAVP-promoted cAMP accumulation in transiently transfected HEK293T cells using cAMP kits to assess the function properties of V2R I324M mutant. As shown in Fig. 4A, the cAMP level of WT-V2R and I324M-V2R had no significant difference in absence of DDAVP. While in the presence of DDAVP, the concentration of cAMP in WT-V2R increased nearly 15-fold than basal state in all set-up concentration groups, and 100 nM DDAVP was chose as the optimum concentration for the cAMP generation. Completely opposite to WT-V2R, there was no obviously response to DDAVP-medicated cAMP generation in the group of I324M-V2R. Even in the case of high dose of DDAVP, the cAMP accumulation was still same as the control (Ctr). The activity of cAMP pathway was not associated with AVP concentration in I324M-V2R group indicate that the mutant perturbed the G protein-dependent cAMP signaling pathway and there was no production of functional receptors. At the same time, we examined the receptor expression from the same samples. Unexpectedly, most mature proteins were synthesized in WT-V2R transfected cells other than in V2R-I324M cells (Fig. 4B). The mature form of V2R can respond to treatment of DDAVP, then activated the cAMP increasement. In short, the loss of mature form in V2R-I324M was an important reason for the inactivation of V2R in cAMP signaling.

### I324M mutant prevented V2R from starting the ERK1/2 signaling pathway under the stimulation of DDAVP

**Figure 5:**
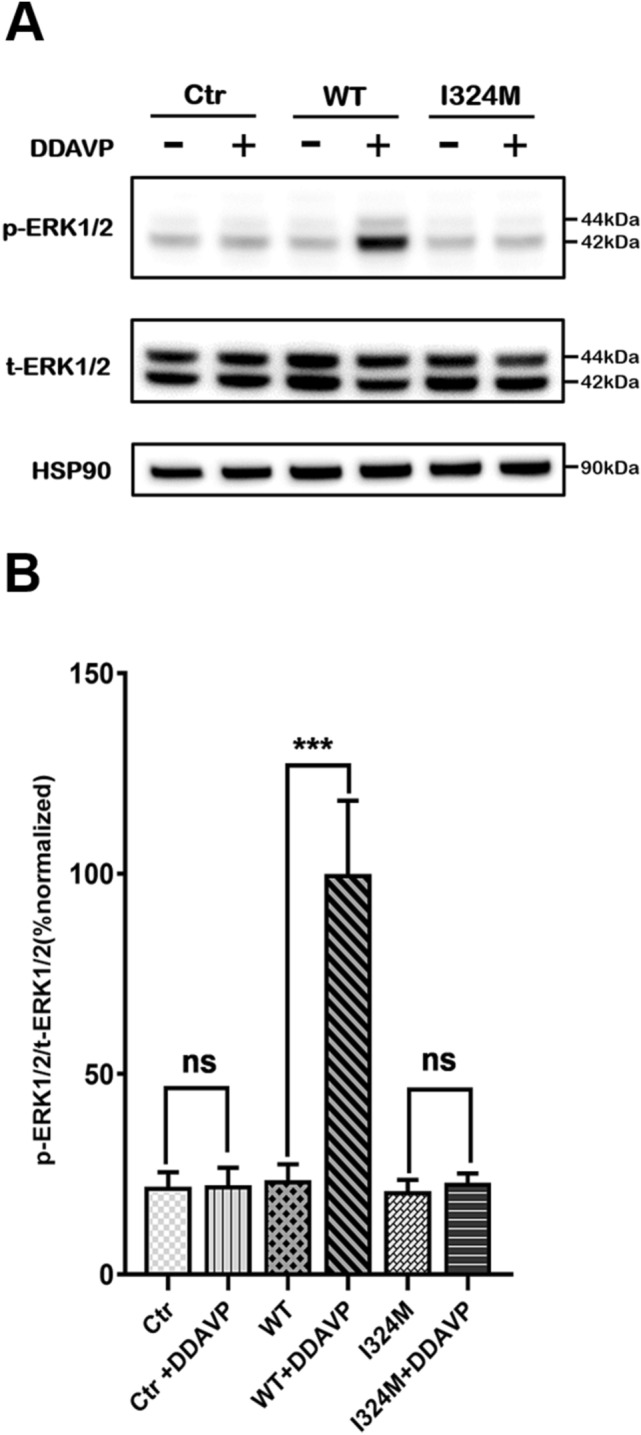
The I324M mutant did not show the activity for initiating the phosphorylation of ERK1/2 signaling pathway. (**A**) HEK293T cells were transiently transfected with plasmid encoding pcDNA3.1 (Ctr), WT-V2R (WT) or I324M-V2R (I324M) and preincubated with or without 1μΜ DDAVP for 15 min before total or phosphorylated ERK1/2 determination was performed by western blotting using anti-ERK1/2 and antiphospho-ERK1/2 rabbit polyclonal antibody, respectively. (**B**) Data was shown as the percentage of p-ERK1/2/t-ERK1/2 resulted from the intensity analysis of protein bands using ImageJ 1.49 and drafted in the GraphPad Prism 7.0. Values were mean ± SEM using One-way ANOVA. ***P < 0.001 (n = 3).

AVP-V2R combination can play a role in promoting water reabsorption by activating G protein- independent MAPK cascade. Consequently, we further measured AVP-induced ERK1/2 phosphorylation (p-ERK) in HEK293T cells either expressing WT-V2R or I324M-V2R that pretreated with 1 μΜ DDAVP for 15 min. The results showed that p-ERK level was similar in absence of DDAVP between WT-V2R and I324M-V2R (Fig. 5A and B, compare lanes 1, 3 and 5). In the stimulation of V2R agonist DDAVP, we found that WT receptor was resulted in a marked ERK1/2 phosphorylation response whereas V2R-I324M was failed to response to DDAVP stimulation (Fig. 5A and B, compare lanes 2, 4 and 6). These results indicated that I324M mutant also terminated the capacity of V2R to elicit the activity of ERK1/2 signaling pathway.

### I324M mutant caused loss of function of V2R was partially rescued upon SR49059 or alvespimycin treatment

**Figure 6:**
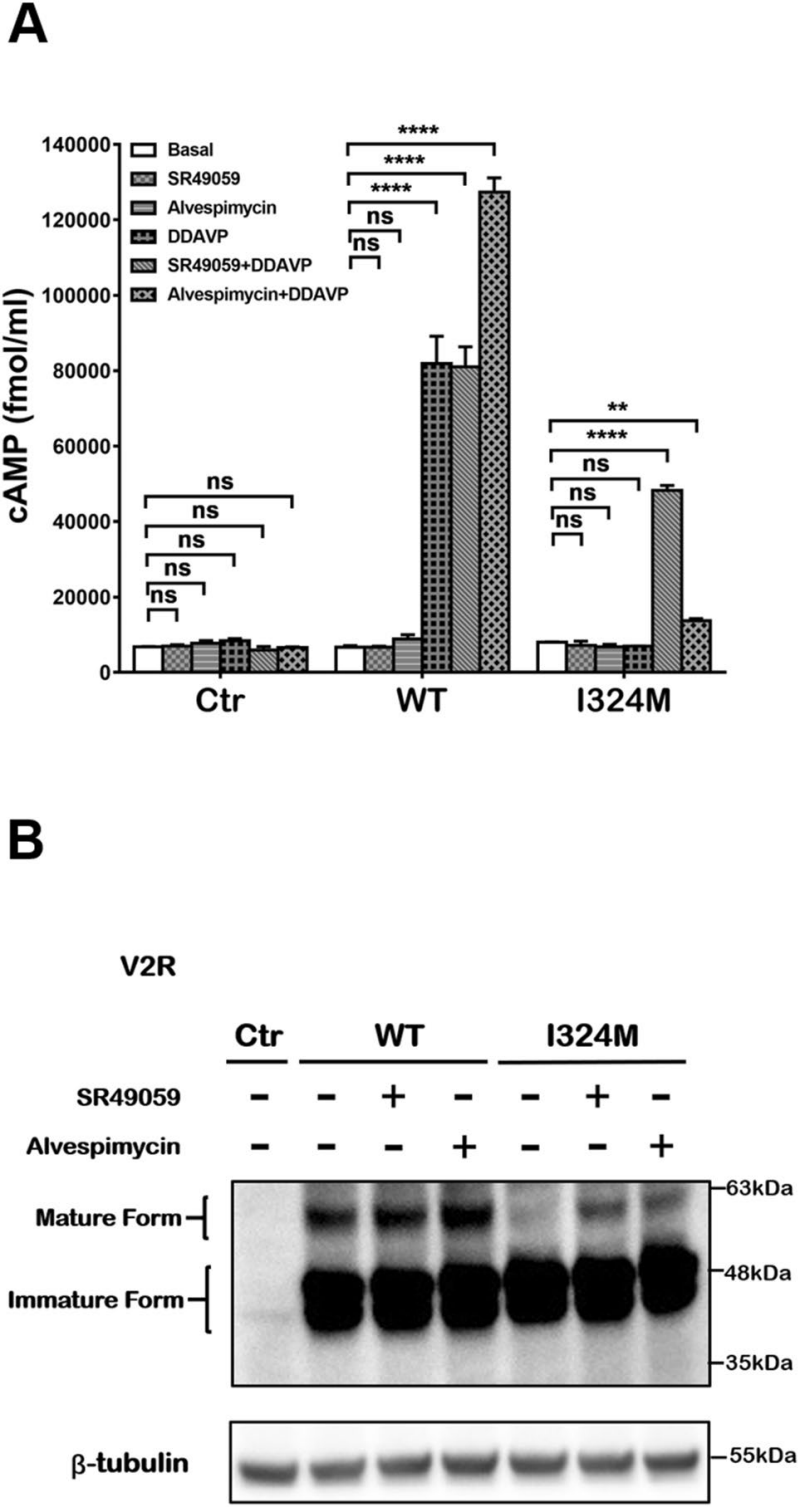
The impaired maturity and cAMP signaling activity of I324M mutant were partially restored by SR49059 and alvespimycin. HEK293T cells were transiently transfected with plasmid encoding pcDNA3.1 (Ctr), WT-V2R (WT) or I324M-V2R (I324M). 24 h after transfection, the cells were incubated with or without SR49059 (1 μM) for 16 h and alvespimycin (1 μM) for 20 h before treated with or without DDAVP (100 nM). (**A**) Results were shown as bar graph and data represent the mean ± SEM using Two-way ANOVA were obtained from three independent experiments. ****P < 0.0001, **P < 0.01. (**B**) V2R expression of WT and I324M groups in the stimulation with or without SR49059 (1 μM) and alvespimycin (1 μM). Representative of 3 separate experiments.

Previous studies have shown that SR49059 is an antagonist of the V1a receptor that can assist some mutant receptor trapped in the intracellular compartment to fold correctly and thus partially restore the functions of the V2R^19^. Alvespimycin is an inhibitor of HSP90 that can increase the synthesis of mature receptors and thereby increasing the ability of V2R signal transduction^21^. Therefore, we selected these two compounds to determine whether they have the similar ability on rescuing the functions of I324M mutant. As shown in Fig. 6A, there was almost the same amount of cAMP in the groups of I324M-V2R and WT-V2R in the absence of DDAVP (basal) or in the presence of SR49059 and alvespimycin. In the case of DDAVP (100 nM) pretreatment, the production of cAMP in WT-V2R group was increased by about 12-fold compare to basal level, while the cAMP level in I324M-V2R group has no any increasement, which was similar to the results as described above (Fig. 4A). Meanwhile cells were treated with DDAVP (100 nM) following incubation with SR49059 or alvespimycin, the cAMP accumulation in I324M-V2R group was increased approximately sevenfold and twofold respectively above non-SR49059 or non-alvespimycin treatment. Furthermore, SR49059 treatment has no significant effect on the group of WT-V2R, while alvespimycin treatment increased cAMP accumulation nearly 1.6-fold compare to the group of AVP (100 nM), which was due to the fact that alvespimycin can also increase the expression of V2R under normal conditions^21^. At the same time, we detected the mature expression of V2R in HEK293T cells upon incubation with SR49059 or alvespimycin. As presented in Fig. 6B, the expression of fully glycosylated receptor (mature form) was obviously increased after treatment with SR49059 or alvespimycin in the group of I324M-V2R compared to basal level (Fig. 6B, compare lanes 5, 6, 7), which may explain the recovery of DDAVP-medicated cAMP response. These results indicated that SR49059 and alvespimycin can play a role in rescuing the receptor maturation and cAMP signaling activity of I324M mutant.

## Discussion

Over recent years, more and more mutations in *AVPR2* gene have been identified in patients with X-linked NDI. Clarifying the molecular defects of these mutations is critical to find potential therapeutic targets for X-linked NDI. In this research, we have functional characterized a clinically relevant *AVPR2* gene mutation (c.972C > G) previously reported in 2016 by our own team, a substitution of isoleucine at position 324 with methionine^22^. Through our studies, we firstly demonstrated the existence of abnormal glycosylation modification and intracellular transport disorder of the I324M mutant receptor. Endoplasmic reticulum (ER) is a strict quality control system, which allowed to be transported out are only properly folded and modified proteins, while misfolded proteins are naturally restricted in it and may target to ER-associated degradation pathway (ERAD)^2, 26, 27^. Subsequently, we performed CHX chase assay to compare protein stability between WT-V2R and I324M-V2R. We showed that the protein degradation rate of I324M-V2R was not affected. The key to determining the transduction of GPCR signals depends on the maturity and appropriate cell localization surface of the membrane receptors^28^. Therefore, we detected the V2R-dependent cAMP and p-ERK1/2 accumulation upon DDAVP stimulation. We found that the functional signaling pathways were inactivated due to maturation and localization errors of I324M-V2R, thus ultimately leading to renal water imbalance.

V2R is an alpha helix receptor, which contains seven transmembrane domains (TMD), four extracellular regions and four intracellular regions^29^. The first and second extracellular regions are often considered to be the ligand binding sites. So, mutations reported in these regions, such as R104C, F105V, R181C, are sure to impair the ligand-receptor binding efficiency. Recently, M311V, M317S and M322S mutants present a reduction of AVP- binding efficiency, indicating that TMD VII is also a potential effect target of ligand^1^. While most of discovered V2R mutations were occurred in membrane spanning and usually exhibited strong retention in the ER^29^. I324M locates on the edge of the TMD VII of V2R, where it forms the special NPXXY motif with N321, P322, W323 and Y325. NPXXY motif is a highly conserved region, where the changes will not only interfere with receptor endocytosis and folding, but also undermine G protein signaling^30, 31^. From previous studies, we know that the binding efficiency of N321Y-V2R, P322S-V2R and Y325F-V2R mutations to ligands was reduced in the case of a similar quantitative cell surface expression compare to WT -V2R, while W323-I324insR-V2R mutation was similar to I324M mutation researched in this paper, both of which lead to receptor misfolding and trapped in ER^31^. For one thing, the conversion of isoleucine (I) to methionine (M) has changes the NPXXY sequence and the additional S-CH3 structure may affect the proper formation of the receptor’s spatial structure, which may be the interpretation for the misfolding and fault localization of I324M-V2R. For another, both of isoleucine and methionine are aliphatic hydrophobic amino acids, so that the replacement between amino acids with identical natures probably has no influence on protein stability and the total protein synthesis. V2R-promoted phosphorylation of ERK1/2 requires the support of β-arrestin. β-arrestin terminates the G protein coupling to participate in receptor endocytosis by competitively binding to V2R with AVP, and at the same time as an adaptor protein to activate ERK1/2^32, 33^. Therefore, in addition to the importance of the NPXXY structure mentioned above, I324M-V2R-mediated silencing of the ERK1/2 pathway may imply that the stability of NPXXY sequence is also essential for V2R-mediate MAPK signaling.

At present, the clinical therapies of X-linked NDI are mainly symptomatic treatment for the clinical manifestation of urinary concentration dysfunction, including fluid administration, low-salt diet, and the application of drugs such as thiazide diuretics and potassium sparing diuretics^19, 34^, the treatment effect however is obviously different among individuals that only alleviates the clinical symptoms in a certain extent. But a complete cure still cannot be achieved which resulted in the life quality of the vast majority of patients is still greatly affected. X-linked NDI is a rare inherited disorder, mostly with V2R single-amino acid abnormalities^35^, which is very difficult to treat due to its resistance to AVP. In recent years, V1a receptor antagonist (SR49059) has been reported as a promising treatment for X-linked NDI. On one hand, SR49059 can decrease 24-h urine volume and water intake and increase urine osmolality in vivo studies. On the other hand, it can increase the cell surface level and the cAMP signaling activity of mutant V2R (R137H, W164S, 185_193del) in vitro studies^20, 36^. In our present studies, we have also shown that the functions of I324M mutant could be rescued to some extent by SR49059, showing as the increasement of mature V2R receptor and cAMP accumulation. However, this compound has no obvious effect on mutants with reduced AVP binding efficiency^20^, which may indirectly indicate that the I324M mutation does not affect the V2R-AVP binding which seems unlike to other mutations in NPXXY region at TMD VII that showed reduced binding affinity. SR49059 has been proved to have a side effect of hepatic toxicity during the second phase of clinical research^20^, so more promising treatments for X-linked NDI need to be explored. At present, alvespimycin is mainly used for the treatment of patients with various kinds of tumors with cells relying on HSP90 activity. Previous studies have demonstrated that alvespimycin has an effect of hyponatremia on patients with advanced solid tumors during the phase I/II study^37^. In response to the results of this literature, Qiong Y et al. believed that the main mechanism explanation of alvespimycin leading to hyponatremia was vasopressin or vasopressin-dependent signaling and shown that alvespimycin can enhance the cell surface level and receptor signaling of WT-V2R in HEK293T cells^21^. In this study, we found that it could also partially restore the maturation and cAMP signaling activity of I324M mutant. The effect of alvespimycin however was found not specific to V2R but common among many GPCRs in recent studies. It was found that alvespimycin can also increase the cell surface expression and fully glycosylated form of A2A adenosine receptor and α2C adrenergic receptor^38, 39^, which may be the main limitation of clinical application of alvespimycin in the future. Therefore, in addition to further exploring more effective treatments for patients carrying I324M mutation, finding novel treatments for various types of V2R mutations is also the top priority of our next research.

To sum up, we have elucidated the molecular basis for the loss of V2R function caused by the I324M mutation and the potential treatments for the functional recovery of I324M-V2R. These findings may provide new ideas for others to understand the molecular mechanism of V2R mutations and develop more promising treatment strategies for patients with X-linked NDI.

## Methods

### Reagents

Dulbecco’s modified eagle medium (DMEM) was obtained from Hyclone (Logan, UT, USA). Penicillin–streptomycin mixture (100X) was from Solarbio Life Sciences (Beijing, China). Fetal bovine serum (FBS) was from Vistech (Austrilian). Phosphate buffer saline (PBS) was from Servicebio (Wuhan, China). Lipofectamine 2000 and 4–12% Bis–Tris Gel were from Thermo Fisher Scientific (Waltham, MA, US). Cycloheximide was from MilliporeSigma (Burlington, MA, USA). Vasopressin analogue desmopressin (DDAVP) was from Absin (Shanghai, China). SR49059, phosphatase inhibitor and proteinase inhibitor were from APExBIO Technology (Houston, TX, USA). Anti-C-Myc rabbit polyclonal antibody was from Immunology Consultants Laboratory (Portland, OR, USA). Anti–protein disulfide isomerase (PDI) mouse monoclonal antibody and cAMP Direct Immunoassay Kits were from Abcam (Cambridge, MA, USA). Anti–protein GM130 mouse monoclonal antibody was from BD Biosciences (Franklin Lakes, NJ, USA). Anti–protein HSP90 mouse monoclonal antibody and anti-protein β-tubulin mouse monoclonal antibody were from Santa Cruz Biotechnology (Dallas, TX, USA). Anti-ERK1/2 rabbit polyclonal antibody and antiphospho-ERK1/2 rabbit polyclonal antibody were from Cell Signaling Technology (Danvers, MA, USA). Alvespimycin was from MedChemExpress (Monmouth Junction, NJ, USA). Endoglycosidase (EndoH), peptide N-glycosidase F (PNGase.F) and O-glycosidase&Sialidase were from Biolabs (New England, MA, USA). cAMP BIOTRAK EIA W/O ACET REG ELISA Kits were from GE Healthcare Life (Germany).

### Expression vectors

Plasmid cDNA encoding human C-terminal Myc-tagged-wild type-V2R (WT-V2R) or Myc-tagged-I324M-V2R (I324M-V2R) subcloned in the pcDNA3.1 vector was generated by TsingKe Biologic Technology, Beijing, China.

### Cell culture and transient expression

HEK293T cells and COS7 cells were cultured in DMEM, supplemented with 10% FBS and 0.1% (v/v) penicillin/streptomycin, in a humidified atmosphere of 5% CO2 at 37 °C. For transient expression, cells were transiently transfected by Lipofectamine 2000 with 0.5 µg (24-well plates), 1 µg (12-well plates) or 2 µg (6-well plates) pcDNA3.1 (as control), WT-V2R or I324M-V2R plasmid cDNA respectively, followed as the manufacturer's protocol. Protein samples were collected after 48 h of transfection and lysed on ice for 20 min with lysis buffer containing Tris–HCl (25 mM), NaCl (100 mM), EDTA (10 mM), 1%TritonX-100, 0.2% sodium deoxycholate, 0.1%SDS, proteinase inhibitors and phosphatase inhibitors for subsequent experiments.

### Western blotting

Prepared protein samples were separated using 4–12% Bis–Tris gradient gels and transferred on the nitrocellulose membranes. Before incubating with primary antibody overnight at 4 °C, the nitrocellulose membranes were blocked in 5% skimmed milk at room temperature for 1 h and then appropriate secondary antibodies conjugated with HRP were used at room temperature for 1 h. Imaging was captured after incubation with the Western ECL Substrate according to the manufacturer’s instructions, using ChemiScope (CliNX science Instruments).

### Immunofluorescence

Transiently transfected COS7 cells were seeded onto glass coverslips and cultured in incubator overnight. Cells were washed 5 min for three times with PBS and fixed with 4% paraformaldehyde for 20 min. Then fixed cells were permeabilized with 0.1% Triton X-100 at room temperature for 10 min and blocked with 5% BSA at room temperature for 1 h. After blocking, the samples were incubating with anti-Myc rabbit polyclonal antibody (1:2000), anti–protein disulfide isomerase (PDI) mouse monoclonal antibody (1:200) or anti-GM130 mouse monoclonal antibody (1:200) respectively at 4℃ overnight. Next day, cells were washed with PBS three times and then incubated with secondary antibody conjugated with different Alexafluor dyes at room temperature for 1 h (away from light). Finally, fluorescent images with a × 40 objective were collected under fluorescent microscope using Axio Imager M2 (Carl Zeiss, Oberkochen, Germany).

### Cycloheximide chase assay

HEK293T cells expressing either WT-V2R or I324M-V2R were cultivated in 12-wells plates. After 24 h of transfection, the cells in each 12-well plate were digested into 4 parts and transferred to the 24-well plate, cultured overnight. Then cells were preincubated with 20 μg/ml cycloheximide (CHX) for 0, 4, 8 or 12 h before protein samples were collected.

### Measurement of cAMP accumulation

For Basal [DDAVP (-)] or DDAVP-promoted cAMP production by using cAMP kits was carried out according to the manufacturer’s introduction of cAMP BIOTRAK EIA W/O ACET REG ELISA Kits. Briefly, HEK293T cells were transiently transfected with Myc-tagged-WT-V2R or Myc-tagged-I324M-V2R. 24 h after transfection, cells were transferred to 96-well plates (160 µl/well), with cell concentrations between 10^4^–10^6^ cells/well and incubated overnight at 37 °C. After additional 24 h, the cells were pretreated with or without 20 μl DDAVP solution (1 nM, 100 nM, 1 μM or 10 μM, respectively) for 15 min. The cells were lysed with 20ul lysis reagent and subjected to cAMP ELISA assay following the manufacturer’s introduction. For functional rescue studies of I324M-V2R, the accumulation of DDAVP-promoted cAMP production in HEK293T cells after incubated with or without SR49059 and alvespimycin was determined by cAMP Direct Immunoassay Kits. Briefly, HEK293T cells were seeded into 6-well plates and transiently transfected with pcDNA3.1 (Ctr), Myc-tagged-WT-V2R or Myc-tagged-I324M-V2R. 24 h after transfection, cells were incubated with SR49059 (1 μM) for 16 h and alvespimycin (1 μM) for 20 h. After additional 20 h, the cells were pretreated with or without DDAVP (100 nM) for 15 min and lysed with 0.1 M HCl, followed by the manufacturer’s introduction.

### Statistical analysis

Data were analyzed using GraphPad Prism 7.0 (https://www.graphpad-prism.cn/) software and values were presented as mean ± SEM. The significance among groups was evaluated using One-way ANOVA or Two-way ANOVA. *P* < 0.01 was considered statistically significant.

## Supplementary Information

Supplementary Information.

## Data Availability

The data generated during and analysed during the current study are available from the corresponding author on reasonable request.
