# Supplementary material for: Functional characterization of a loss-of-function mutant I324M of arginine vasopressin receptor 2 in X-linked nephrogenic diabetes insipidus
Source: Sci Rep. 2021 May 26;11:11057. doi: 10.1038/s41598-021-90736-z (PMC8154955; doi:10.1038/s41598-021-90736-z)

Authors:

Lixia Wang, Weihong Guo, Chunyun Fang, Wenli Feng, Yumeng Huang, Xiaona Zhang, Ming Liu, Jingqiu Cui

Supplementary Information 1: (A) The full-length blot/gel of manuscript's Figure 1A and Figure 1B. (B) The full-length blot/gel of manuscript's Figure 1C

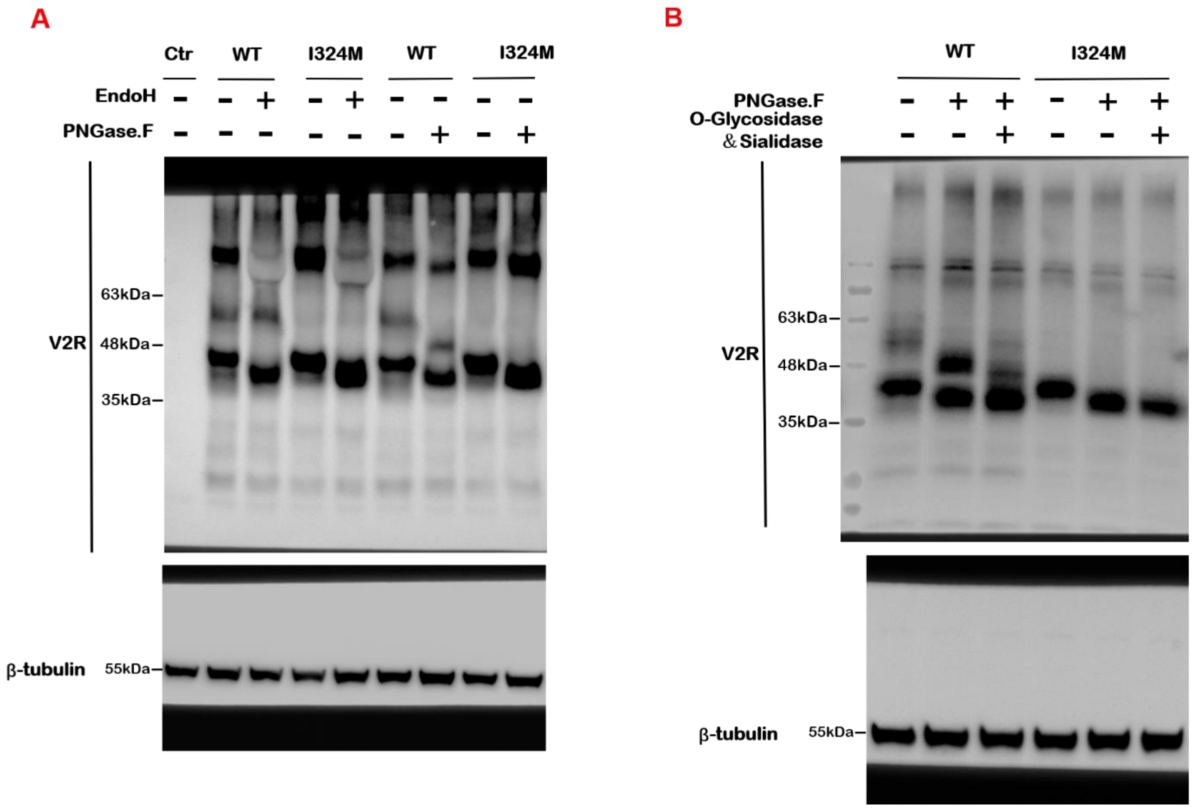

Supplementary Information 2: The full-length blot/gel of manuscript's Figure 3A

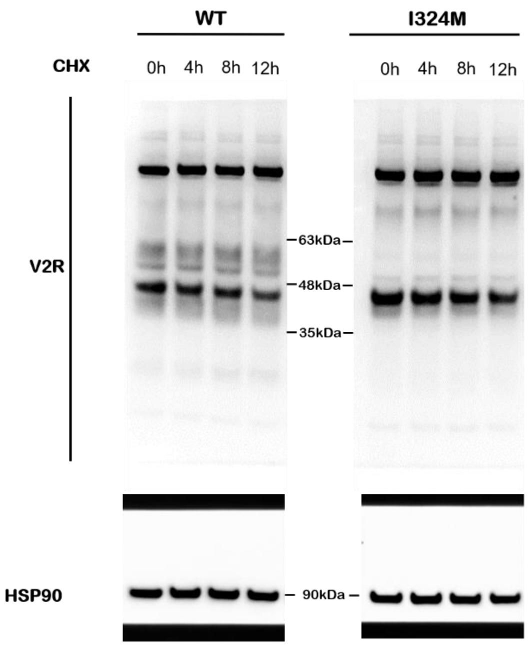

Supplementary Information 3: The full-length blot/gel of manuscript's Figure 4B (Indicated in the red box)

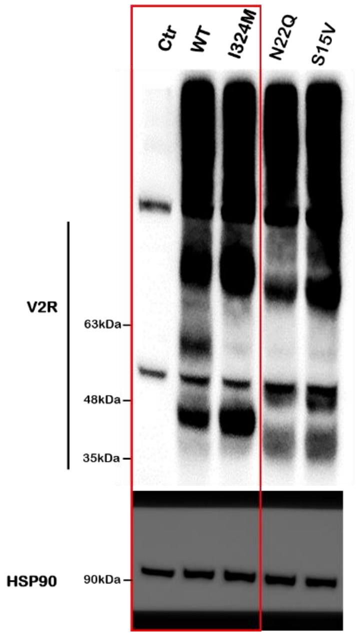

Supplementary Information 4: The full-length blot/gel of manuscript's Figure 5A

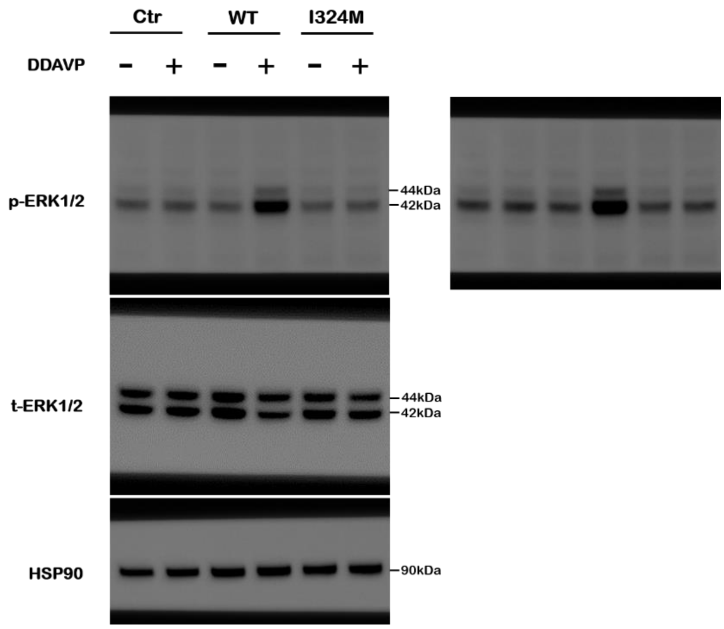

Supplementary Information 5: The full-length blot/gel of manuscript's Figure 6B

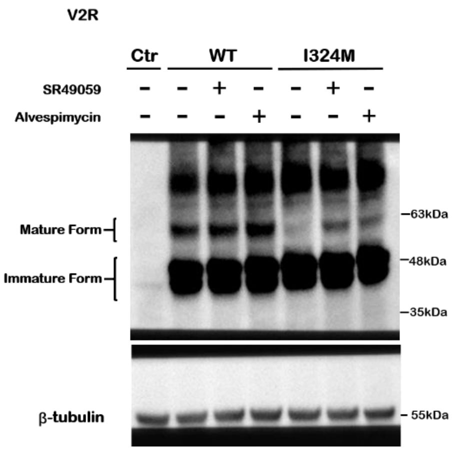

Supplement: Supplementary file 1 — Supplementary Information. [file 41598_2021_90736_MOESM1_ESM.pdf]
